# Cumulative Impact of Environmental Pollution and Population Vulnerability on Pediatric Asthma Hospitalizations: A Multilevel Analysis of CalEnviroScreen

**DOI:** 10.3390/ijerph16152683

**Published:** 2019-07-27

**Authors:** Emanuel Alcala, Paul Brown, John A. Capitman, Mariaelena Gonzalez, Ricardo Cisneros

**Affiliations:** 1 Department of Public Health, School of Social Sciences, Humanities, and Arts, University of California, Merced, CA 95343, USA; 2 Central Valley Health Policy Institute, College of Health and Human Services, California State University, Fresno, CA 93740, USA

**Keywords:** screening tool, environmental justice, air pollution, pediatric asthma, multilevel analysis

## Abstract

The CalEnviroScreen created by the Office of Environmental Health Hazard Assessment, Sacramento, USA, is a place-based dataset developed to measure environmental and social indicators that are theorized to have cumulative health impacts on populations. The objective of this study was to examine the extent to which the composite scores of the CalEnviroScreen tool are associated with pediatric asthma hospitalization. This was a retrospective analysis of California hospital discharge data from 2010 to 2012. Children who were hospitalized for asthma-related conditions, were aged 0–14 years, and resided in California were included in analysis. Rates of hospitalization for asthma-related conditions among children residing in California were calculated. Poisson multilevel modeling was used to account for individual- and neighborhood-level risk factors. Every unit increase in the CalEnviroScreen Score was associated with an increase of 1.6% above the mean rate of pediatric asthma hospitalizations (rate ratio (RR) = 1.016, 95% confidence interval (CI) = 1.014–1.018). Every unit increase in racial/ethnic segregation and diesel particulate matter was associated with an increase of 1.1% and 0.2% above the mean rate of pediatric asthma, respectively (RR = 1.011, 95% CI = 1.010–1.013; RR = 1.002, 95% CI = 1.001–1.004). The CalEnviroScreen is a unique tool that combines socioecological factors and environmental indicators to identify vulnerable communities with major health disparities, including pediatric asthma hospital use. Future research should identify mediating factors that contribute to community-level health disparities.

## 1. Introduction

Asthma is the most common chronic health condition in children associated with hospitalization and causes severe life disruption that can be prevented with appropriate care and services [1,2]. Estimated annual costs for pediatric asthma-related hospitalizations were $1.59 billion in 2009 [3]. Multiple pathways contribute to asthma exacerbations ranging from behavioral and social to environmental, including cigarette smoke, genetic predisposition, diet, poor housing conditions, and indoor and outdoor air pollution, among other factors [4,5,6,7,8]. Despite major advances in medicine, treatment, and program implementation to combat the aforementioned pathways, pediatric asthma health care utilization and costs have been shown to rise, suggesting unexplored pathways [1,9]. Furthermore, the disparity in rates of pediatric asthma exacerbations, severity, and poor quality of care across socioeconomic status and racial/ethnic minorities have been shown to widen [2]. 

Researchers have documented the geographic disparity of the burden of asthma and how social characteristics of neighborhoods such as poverty, racial/ethnic segregation, psychosocial stress, and collective efficacy contribute to asthma [10,11,12]. Beyond social characteristics of neighborhoods, studies have identified environmental pollutants associated with asthma to suggest that particularly vulnerable (e.g., low-income, youth, racial/ethnic minorities) populations demonstrate a greater health response to environmental exposures [13,14]. In response to the scientific evidence suggesting that cumulative exposure to environmental pollutants has detrimental health effects on socially deprived populations, frameworks and screening tools have been developed to identify neighborhoods that are disadvantaged and overburdened with multiple social stressors and environmental pollutants [15,16,17,18,19,20].

The CalEnviroScreen, created by the Office of Environmental Health Hazard Assessment, Sacramento, USA, is one such screening tool with major societal and public health implications. It was collaboratively developed by the California Environmental Protection Agency (CalEPA) and the Office of Environmental Health Hazard Assessment (OEHHA) to identify communities disadvantaged by a cluster of social and environmental injustices to inform governmental agencies on where to allocate resources toward improving the environment and the health of the people. A central aim of the tool is to promote economic prosperity and to reduce public health disparities clustered in a geographic area. However, little is known about the extent to which the CalEnviroScreen tool and its composite scores do in fact identify communities with major public health concerns.

This exploratory analysis investigates associations between the CalEnviroScreen composite scores, as well as between levels of separate indicators of the CalEnviroScreen screening tool, with pediatric asthma hospitalization rates, to examine the extent to which these measures are associated with pediatric asthma hospitalization.

## 2. Materials and Methods

Patient discharge data (hospital discharge), from 2010 to 2012, from California’s Office of Statewide Health Planning and Development (OSHPD) were analyzed because these years closely align with the measures within the CalEnviroScreen tool. Hospitals in the state of California are required to report all visitation records to OSHPD. The sample population of children aged 0 to 14 resided in a zip code within California and the expected source of payment was Medi-Cal (Medicaid in California) or private insurance. Children older than 14 years of age were omitted from analyses because these asthma cases made up less than 1% of all pediatric patients hospitalized with asthma. Hospital discharge date and the CalEnviroScreen were linked by the patients’ zip code of residence.

### 2.1. Composite Scores of the CalEnviroScreen

The CalEnviroScreen screening tool was computed by the Office of Environmental Health Hazard Assessment (OEHHA) and has three key composite scores that are used in this study: the CalEnviroScreen score (CES), population characteristics score (PCS), and pollution burden score (PBS). The CES ranged from 0 to 100 and was the product of the PCS and PBS representing cumulative disadvantage of social and environmental risks at the zip code level. The PCS ranged from 0 to 10 and was a composite score of socioeconomic, demographic, and health indicators (i.e., educational attainment, linguistic isolation, poverty, racial/ethnic minorities (% nonwhite), children and older adults, asthma, low birth weight). The PCS ranged from 0 to 10 and was the composite score of 11 environmental pollutants (i.e., ozone, particulate matter 2.5, diesel particulate matter (PM), pesticide use, toxic releases, traffic density, cleanup sites, groundwater threats, hazardous waste, impaired water bodies, and solid waste sites). Diesel particulate matter (PM) was measured as the spatial distribution of gridded diesel PM emissions from onroad and nonroad sources for a 2012 summer day in July (kg/day). Diesel PM emissions data was collected from the California Air Resources Board. Poverty was the percent of the population living below two times the federal poverty level (five-year estimate, 2011–2012). Linguistic isolation was the percent of limited-English-speaking households (2011–2015). The racial/ethnic minorities indicator was measured as the percent of the population that was nonwhite or Hispanic/Latino including all people in the U.S. Census who identified a race other than white or who identified as Hispanic, Latino, or of Spanish origin. In this study, all composite scores and indicators were centered around the mean values prior to analyses. A full description of methodology to compute the composite scores and data sources used to create the CalEnviroScreen can be found in the supplementary material (Table S1).

### 2.2. Person-Level Indicators

Individual-level indicators included age, sex, race/ethnicity, and insurance coverage. Dummy coding was used for individual-level predictors. Age was coded to include children younger than 5; 5 to 9; and 10 to 14 (reference group). Sex was coded for boys and girls (reference group). The five racial/ethnic groups were black/African-American, Latino/Hispanic, Asian, Other race/ethnicity, and non-Hispanic white (reference group). Insurance coverage was dichotomized into Medi-Cal (Medicaid in California) and private (reference group) where all other types of coverage were omitted from the analysis.

The project number is 12-06-0430. The Committee for the Protection of Human Subjects (CPHS) initially approved this project on 2 August 2013. Annually, the project is renewed and is currently active. All subject-related data for this study was collected from the Office of Statewide Planning and Development (OSHPD) and was deidentified prior to acquisition.

### 2.3. Outcome of Interest

The outcome of interest was counts of potentially preventable asthma-related hospitalizations. Prevention quality indicators (PQIs) from the Agency of Healthcare Research and Quality (AHRQ) were used as criteria in identifying potentially preventable asthma-related primary diagnoses. International Classification of Diseases, 9th Revision, Clinical Modification (ICD-9-CM) codes were used to identify asthma events and included: 49300, 49301, 49302, 49311, 49312, 49320, 49321, 49322, 49382, 49390, 49391, and 49392. These ICD-9-CM codes are consistent with Lu who adapted these codes for use in a pediatric population [21,22].

### 2.4. Population at Risk

Similar to previous studies, we estimated the number of children at risk for hospitalization by regression [12,23]. The estimated number of children at risk was used as the denominator to calculate population rates of hospitalization as well as to use as an offset variable in regression analyses. To model population rates of asthma, 60 cells per zip code were used as population categories; each cell had an event rate estimated. The cells were categorized by race/ethnicity (non-Hispanic white, black/African-American, Latino, Asian, and Other race/ethnicity), age (0–4, 5–9, and 10–14 years), sex (female and male), and insurance coverage (Medi-Cal and private) that had 5, 3, 2, and 2 levels, respectively. The numerator in each cell was the count of asthma events in a specific age, sex, racial/ethnic, and insurance category, and the denominator was the estimated count of the population at risk. The population at risk was estimated by using ordinary least square regression to predict the proportion of children eligible for Medi-Cal in California at the zip code level by percentages of categories in age, sex, race/ethnicity, and the proportion of individuals living two times below 138% of the poverty level, *F*(13, 1376) = 93.9, *p* < 0.001, R^2^ = 0.47. The same regression model was used to predict the population that is eligible for private insurance coverage, *F*(13, 1376) = 70.7, *p* < 0.001, R^2^ = 0.40. We then multiplied the predicted proportions of eligibility by the number of children living in a zip code to compute the population at risk (denominator of rate).

### 2.5. Statistical Analyses

We conducted summary statistics for the sample population by sex, age, race/ethnicity, and insurance coverage shown in Table 1. Descriptive statistics of the composite scores and indicators of the CalEnviroScreen tool as well as for the outcome of interest are displayed in Table 2. These analyses were conducted in STATA 14 (StataCorp LLC, College Station, USA).

Due to the nested nature of these data, children living within zip codes, hierarchical generalized nonlinear modeling was used to account for clustered data at multiple levels. It was appropriate to use Poisson-based models for parameter estimation due to the discrete, non-negative, and rare nature of the outcome of interest, asthma hospitalizations. We specified the general pediatric population in each zip code as the offset (population at risk) in each model. These methods have been used in previous research investigating rates of hospitalizations and geographical analyses [23,24]. We tested varying-intercept models to examine the CalEnviroScreen’s capacity for prediction at the population level [25]. Model 0 examined if asthma hospitalizations significantly varied at the zip code level, after adjusting of individual-level risk factors. Model 1 introduced the composite CalEnviroScreen score. Model 2 omitted the composite CalEnviroScreen score and tested both components, pollution burden and population characteristics score. The final model, Model 3, represents all of the environmental pollutant and population characteristic indicators that remained significant after adjusting for all CalEnviroScreen indicators, beyond individual-level risk factors. Multilevel analyses were conducted in HLM7. HLM7 provides several types of output; it was appropriate to interpret population-average models with robust standard errors [25]. 

## 3. Results

Table 1 illustrates the frequency, proportion, and hospitalization rate for asthma-related diagnosis by demographic characteristics for children. Boys (53%) used hospital services more than girls did (47%) during this period. The youngest children (43%) used the majority of hospital services with children aged 5 to 9 (30%) using services at a similar proportion to children aged 10 to 14 (27%). Whites (33%) and Latinos (31%) composed the majority of hospital discharges and African Americans (11%) were the smallest proportion of hospital discharges. A greater proportion of privately (55%) insured children were discharged compared to children covered by Medi-Cal (45%). Population-level rate estimates were generally as expected; African-American children had more than three times the risk for hospitalization than white children (14 vs. 4 per 10,000, respectively). Similarly, children on Medi-Cal were about three times at risk for hospitalization than privately insured children (10 vs. 3 per 10,000, respectively).

There were 1769 zip codes included in the CalEnviroScreen. Among all the zip codes in the screening tool, 901 valid zip codes matched a patient’s zip code of residence and were included in the analyses. Table 2 shows descriptive statistics on the CalEnviroScreen indicators as well as on counts of asthma discharges and population estimates of children at risk (offset variable). The mean rate of pediatric asthma hospital discharges across zip codes was 22 per 10,000 children at risk.

A null model (varying-intercept) indicated that mean rates of asthma hospitalizations varied significantly by zip codes (*b* = –5.94), translating into a risk ratio (RR) = 0.0026 (0.002, 0.003), or 26 per 10,000 in the population at risk. This finding suggested that multilevel modeling was appropriate. Table 3 displays the estimated coefficients and standard errors for asthma hospitalizations for three hierarchical generalized nonlinear regression models with only varying-intercepts. In Model 0, we adjusted for level-one indicators indicating that across zip codes, the mean rate of asthma hospitalizations for children (i.e., girl, white, age 10 to 14, private insurance) remained significant (*b* = −7.177), translating into a risk ratio (RR) = 0.0007 (0.001, 0.001), or 7 per 10,000 in the population at risk. Across zip codes, the risk for hospitalization was, on average, increased if the children were on Medi-Cal exp(1.412) = RR 4.10 (3.965,4.256), black/African-American exp(0.382) = RR 1.46 (1.391, 1.547), younger than five years of age exp(1.292) = RR 3.64 (3.519,3.770), or age five to nine exp(0.246) = RR 1.27 (1.244,1.315). Children were at lower risk for asthma hospitalizations if they were Latino exp(−0.226) = RR 0.79 (0.773,0.822), Asian exp(−0.286) = RR 0.750 (0.715,0.788), or other race/ethnicity exp(−0.910) = RR 0.40 (0.359,0.451). After adjusting for level-one factors, the variance components at the zip code level remained significant (t00 = 0.171, X^2^ 17266, *p* < 0.001). That is, significant differences in mean rates of asthma hospitalizations existed between zip codes, after accounting for individual-level characteristics. 

Model 1 introduced the CalEnviroScreen Score (CES) to the model. CES was positive (*b* = 0.016) and significant (*p* < 0.001). For every unit increase in CES, the risk ratio for mean hospitalizations would be expected to increase by 1.016 (1.014, 1.018) times the mean rate. Thus, if we compare a zip code with the mean CES value (24) to zip code with a CES one standard deviation above the mean (36) that are otherwise similar, we would expect an (0.016) × (12) = 0.19, RR 1.21, translating to 9 per 10,000 in population at risk. Model 2 examines the two composite scores that compose the CES, the pollution burden score (PBS) and the population characteristics score (PCS). The effect of PCS (*b* = 0.103, *p* = 0.009) was more than twice as large as the PBS (*b* = 0.016, *p* = 0.008). Individual-level effects remained similar to Model 0 for Models 1 to 3.

Table 4 displays results of the final model. In developing the final model, all pollutant indicators that are indexed in the PBS were examined. A saturated model indicated that ozone, diesel PM, pesticide use, toxic releases (natural log), and ground water waste sites were significant and positively associated with asthma, beyond person-level factors. Then, all of the population indicators that are indexed in the PCS were examined. A saturated model including all population characteristic indicators suggested that % linguistic isolation, % poverty, and % racial/ethnic minorities were significant and associated with asthma, beyond person-level factors. Model 3 is the final model of the analysis and illustrates significant pollutants and population characteristics, beyond person-level factors. Diesel PM (*b* = 0.002, *p* < 0.001) was the only pollutant that remained significant after adjusting for % linguistic isolation, % poverty, and % racial/ethnic minorities at the zip code level. Increasing levels of linguistic isolation were significantly associated with lower zip-code-level asthma hospitalization (*b* = −0.013, *p* = 0.002). Conversely, increasing levels of poverty and racial/ethnic minorities were associated with greater zip-code-level asthma hospitalization (*b* = 0.003, *p* < 0.001; *b* = 0.011, *p* < 0.001, respectively).

## 4. Discussion

The aim of this study was to investigate the extent to which the CalEnviroScreen could identify populations and communities with the highest burden of pediatric asthma hospitalization rates. The composite scores (i.e., CalEnviroScreen score, pollution burden score, population characteristics score) were examined, as well as the separate indicators within the composite scores. Our main finding is that the composite score of the CalEnviroScreen tool does identify communities with the highest rates of asthma hospitalizations and communities with high concentrations of diesel PM, poverty, and racial/ethnic minorities were most strongly associated. These findings contribute to the discussion on the CalEnviroScreen’s capacity to identify public health issues in disadvantaged communities as well as its use in the broader landscape of measuring the impacts of social and environmental inequities that are geographically dispersed. Although this article does not measure the potential interactions of chemicals in the environment or the synergistic effects they may have on populations, understanding how exposure to additive social and environmental inequities impact pediatric asthma has major public health implications [26,27].

Although Greenfield, Rajan, and McKone conducted an analysis of the CalEnviroScreen, examining the extent to which the tool identified disease burden, we build upon the literature by examining a specific disease outcome among children and by disentangling person-level effects from community-level effects [28]. Our methodology allows for an interpretation of community-level effects adjusting for person-level risk factors for asthma morbidity, a strategy that has been advocated for within the public health literature [29,30].

At the zip code level, increased levels of linguistic isolation were associated with a reduction in asthma hospitalizations. The clustering of immigrants, lower educational attainment, and households with non-English-speaking adults may explain the reduced rates of health care utilization [31]. Previous studies have used household linguistic isolation to determine sociocultural barriers to accessing health care services and show that this is an independent barrier to health care access in comparison to socioeconomic status and health care needs [32]. Recent studies have found neighborhood-level associations with asthma and are similar to our finding. For example, several studies in urban Chicago have found the clustering of foreign-born populations provides protective effects on asthma [10,33]. Moreover, collective efficacy and social cohesion have been theorized as a mechanistic pathway from neighborhood to health [33,34]. The Latino population in California is primarily of Mexican descent, aligning well with the Latino population of Chicago [11]. To the best of our knowledge, the only study that specifically investigated neighborhood-level effects of linguistic isolation on pediatric asthma did not find an association [35]. This study did not find any associations that were significant between census-tract-level factors and the likelihood of children ever being diagnosed with asthma. This study implemented a two-stage hierarchical modeling method that does not appropriately weight parameter estimates by group size; therefore, standard errors tended to be large. It should also be noted that a different set of factors contributes to asthma diagnosis in comparison to preventable asthma hospitalizations. Due to the limitations of our current study, it cannot be ruled out that children living in communities with high concentrations of linguistic isolation or immigrant families are less likely to demonstrate symptoms of asthma or be diagnosed with asthma. The findings of the current analysis could reflect this phenomenon. 

Increased levels of poverty and racial/ethnic segregation were associated with increased mean rates of asthma hospitalizations. Concentrated poverty has been demonstrated to harbor the conditions for severe asthma exacerbations [12,36,37,38,39,40,41]. There is evidence that racial/ethnic minorities are segregated into communities with greater social and environmental burden [17,27,42,43]. The sociopolitical historical events that shaped communities of color and placed multiple sources of pollution differ across regions within California [19,42,43]. However, the framework of allostatic load, the adverse physiological response to chronic stress exposure, may provide insight into our finding of racial/ethnic segregation and on how asthma exacerbations may be induced by chronic exposure to stress [43,44,45,46]. In an analysis of the Study of Asthma, Genes, and the Environment (SAGE) cohort, researchers found that increased levels of allostatic load were associated with greater odds of developing asthma in comparison to children exposed to lower allostatic load [47].

The geographical distribution of diesel particulate matter was associated with increased mean rates of asthma hospitalizations. Our data suggested that the relationship between diesel particulate matter and asthma hospitalization was strongest in zip codes near highway 99 in the San Joaquin Valley. There is a large body of literature linking traffic-related air pollution to asthma exacerbations [48,49,50]. The UK’s Committee on the Medical Effects of Air Pollutants have identified four plausible mechanistic pathways from air pollution exposure to severe asthma exacerbation: oxidative stress and damage, airway remodeling, inflammatory pathways and immunological responses, and enhancement of respiratory sensitization to aeroallergens. Children’s allergic inflammation and immunological responses can be enhanced in the presence of diesel exhaust [49,50,51]. Furthermore, vehicular emissions have been shown to impact pulmonary function in children [13,46,47].

This study demonstrates that social factors, at the individual level and community level, are largely associated with asthma morbidity even after adjusting for environmental pollutants. Although diesel particulate matter was the only environmental pollutant that remained significant after adjusting for population characteristics, there is potential for misclassification errors to exist in these data. Methodology for measuring environmental pollutants are not well established, especially in comparison to population characteristic indicators. This would indicate that coefficient estimates for environmental pollutants in this study, including parameters presented for diesel particulate matter, may be underestimated and population characteristics may be overestimated. In developing the multilevel models, all environmental pollutants measured by the CalEnviroScreen were singularly significant in their association with asthma hospitalizations. It was only after adjusting for other zip-code-level factors that their effects were nonsignificant.

### Limitations

We were only able to link 47% of CalEnviroScreen to hospital records. The values of the zip codes from the CalEnviroScreen that were retained tended to be greater than the values that were dropped. This may have biased our results because we were not able to incorporate the lower spectrum of the CES scores. Although this reduces the total amount of variability in our analysis, our parameter estimates of asthma disparities are conservative due to the omission of lower CES scores as well as the reduction in indicator variability. Our data are limited in the ability to account for well-established, in-home triggers of asthma such as smoking, mold, and other allergens. The Latino/Hispanic population in California is largely Mexican and Mexican-American, and has consistently been shown to have lower asthma prevalence compared to other racial/ethnic groups. This may present a confounding effect in our ability to identify risk factors for preventable pediatric asthma hospitalization.

## 5. Conclusions

The CalEnviroScreen does identify disadvantaged communities with disparities in pediatric asthma hospitalizations, beyond the person-level risk factors accounted for in this study. At the state level, policy addressing diesel particulate matter, barriers to access due to linguistic isolation, concentrated poverty, and racial/ethnic segregation could be implemented to attenuate asthma disparities and provide hospital services to those who need them. However, the total score does not provide insight on the historical, contextual, or environmental risks occurring at the local level because regions and neighborhoods within California have been shaped and are impacted by different environmental pollutants and populations. Local communities need further data to inform decision making. 

## Figures and Tables

**Table 1:** Frequency, percentage, and rate of asthma-related hospitalization by characteristic (n = 21,264), 2010–2012, California.

Characteristic	n	%	Hospitalization Rate/10,000 in Population
Sex			
Female	10,074	47.4%	4.7
Male	11,190	52.6%	4.7
Age Group			
Younger than 5 years of age	9178	43.2%	5.6
5 to 9 years of age	6290	29.6%	4.6
10 to 14 years of age	5796	27.2%	3.8
Race/Ethnicity			
White	7072	33.3%	4.2
African-American	2253	10.6%	14.1
Latino	6620	31.1%	3.6
Asian	2634	12.4%	5.2
Other race/ethnicity	2685	12.6%	7.8
Insurance			
Medi-Cal	9510	44.7%	10.2
Private Insurance	11,754	55.3%	3.3

**Table 2:** Descriptive statistics across zip codes (n = 901) of CalEnviroScreen version 1 and outcome of interest, 2010–2012, California.

Indicator	Mean	SD	Min	Max
CalEnviroScreen score	24.01	11.83	2	66
Pollution burden score	4.8	1.35	1	9
Ozone (ppm)	0.11	0.19	0	1.29
Particulate matter 2.5 (µg/m^3^)	11.03	2.92	4	21
Diesel particulate matter (kg/d)	12.11	12.88	0	125
Pesticide use (ln lb/square mile)	1.9	2.71	0	10.14
Toxic releases (ln toxicity weighted lb/y)	5.51	6.07	0	20.06
Traffic density (vehicle-km per h/km)	6.76	0.91	0	8.47
Cleanup site (ln weighted sum)	2.49	1.59	0	6.18
Groundwater threats (ln weighted sum)	3.81	1.5	0	8.42
Hazardous waste sites (weighted sum)	2.99	4.26	0	59
Impaired water bodies (sum of pollutants)	5.01	5.44	0	32
Solid waste sites (weighted sum)	5.63	7.14	0	57
Population characteristics score	4.94	1.66	1	10
Children and older adults (%)	25.57	4.14	9	59
Asthma emergency department (visits per 10,000/y)	42.18	26.31	7	312
Low birth weight (%)	6.64	1.27	2	15
Educational attainment (%)	16.55	12.38	1	75
Linguistic isolation (%)	8.59	7.26	0	48
Poverty (%)	30.7	15.54	4	91
Race/ethnicity (% nonwhite)	50.7	21.94	10	98
Asthma hospitalization count	4.7	8.25	1	200
Population at risk	2127	3041	36	39,459

Note. Standard deviation = SD. Pounds = lb.

**Table 3:** Rates of asthma hospitalizations estimated by hierarchical generalized nonlinear regression models by zip-code-level and individual-level indicators, 2010–2012, California.

Indicator	Model 0		Model 1		Model 2	
	*b*	SE	*b*	SE	*b*	SE
Zip code level (n = 901)						
Intercept	−7.177 ***	0.023	−7.192 ***	0.022	−7.192 ***	0.022
CalEnviroScreen Score	-	-	0.016 ***	0.001	-	-
Pollution Burden Score	-	-	-	-	0.045 ***	0.009
Population Characteristics Score	-	-	-	-	0.103 ***	0.008
Person level (n = 21,264)						
Boys	0.221 ***	0.008	0.221 ***	0.008	0.221 ***	0.008
Younger than 5 years of age	1.292 ***	0.017	1.292 ***	0.017	1.292 ***	0.017
5 to 9 years of age	0.246 ***	0.014	0.246 ***	0.014	0.246 ***	0.013
Black/African-American	0.382 ***	0.027	0.373 ***	0.024	0.371 ***	0.024
Latino/Hispanic	−0.226 ***	0.015	−0.236 ***	0.015	−0.238 ***	0.015
Asian	−0.286 ***	0.024	−0.291 ***	0.024	−0.292 ***	0.024
Other race/ethnicity	−0.910 ***	0.058	−0.920 ***	0.051	−0.921 ***	0.051
Medi-Cal	1.412 ***	0.018	1.407 ***	0.016	1.406 ***	0.017
LL function at iteration 2	−5.24 × 10^4^		−5.13 × 10^4^		−5.14 × 10^4^	
Variance component	0.171 ***		0.142 ***		0.137 ***	

*** *p* < 0.001. ** *p* < 0.01. * *p* < 0.05. Standard error = SE.

**Table 4:** Final model of rates of asthma hospitalizations estimated by hierarchical generalized nonlinear models by zip-code-level and individual-level indicators, 2010 2012, California.

Indicator	Model 3	
	*b*	SE
Zip code level (n = 901)		
Intercept	−7.195 ***	0.022
Diesel PM	0.002 *	0.001
% Linguistic Isolation	−0.013 ***	0.002
% Poverty	0.003 **	0.001
% Racial/Ethnic Minorities	0.011 ***	0.001
Individual level (n = 21,264)		
Boys	0.221 ***	0.008
Younger than 5 years of age	1.293 ***	0.016
5 to 9 years of age	0.246 ***	0.013
Black/African-American	0.364 ***	0.024
Latino/Hispanic	−0.242 ***	0.016
Asian	−0.302 ***	0.023
Other race/ethnicity	−0.926 ***	0.051
Medi-Cal	1.407 ***	0.017
LL function at iteration 2	−5.10 × 10^4^	
Variance component	0.130 ***	

*** *p* < 0.001. ** *p* < 0.01. * *p* < 0.05. Particulate matter = PM. Log Likelihood = LL.

## Supplementary Materials

The following are available online at https://www.mdpi.com/1660-4601/16/15/2683/s1, Table S1: CalEnviroScreen Indicators at the Zip Code Level, Description and Data Source.
