# Supplementary material for: Cumulative Impact of Environmental Pollution and Population Vulnerability on Pediatric Asthma Hospitalizations: A Multilevel Analysis of CalEnviroScreen
Source: Int J Environ Res Public Health. 2019 Jul 27;16(15):2683. doi: 10.3390/ijerph16152683 (PMC6696276; doi:10.3390/ijerph16152683)
Supplement: Supplementary file 1 [file ijerph-16-02683-s001.pdf]

Supplementary Materials

Table S1. CalEnviroScreen Indicators at the Zip Code-level, Description and Data Source.

Indicator	Description and Data Source
CalEnviroScreen score	The average percentile ranks of the indicators below, weighted by the highest ranking score and multiplied by 10
Pollution burden score	The average of percentile rank scores of exposure indicators and the environmental effects indicators one-half weighted, weighted by the highest ranking score and multiplied by 10
Ozone (ppm)	Portion of the daily maximum 8-h ambient concentration over the federal standard (2007–2009 CARB)
Particulate matter 2.5 (μg/m^3)	Annual mean ambient concentration (2007–2009 CARB)
Diesel particulate matter (kg/d)	Emissions from on-road and nonroad sources for a 2010 summer day in July (CARB)
Pesticide use (ln lb/square mile)	Selective active ingredients used in production agriculture (2009–2010 California Department of Pesticide Regulation)
Toxic releases (ln toxicity weighted lb/y)	Releases to air or water (2008–2010 Toxics Release Inventory, US Environmental Protection Agency)
Traffic density (vehicle-km per h/km)	Traffic volume by road length within 150 meters of zip code boundary (2004 CEHTP, CDPH)
Cleanup site (ln weighted sum)	Cleanup sites weighted by site type and status (2013 DTSC EnviroStor database)
Groundwater threats (ln weighted sum)	Potential contamination sources and monitoring wells, weighted by site type and status (2013 SWRCB GeoTracker database)
Hazardous waste sites (weighted sum)	Permitted hazardous waste facilities and generators, weighted by waste type and volume (2013 DTSC EnviroStor database)
Impaired water bodies (sum of pollutants)	Number of pollutants across water bodies designated as impaired (2010 SWRCB 303(d) List of Impaired Water Bodies)
Solid waste sites (weighted sum)	Solid waste facilities, operations, and disposal sites, weighted by site type and status (2013 SWIS and CIA Disposal Sites Program, Department of Resources, Recycling and Recovery)
Population characteristics score	The average percentile ranks of the indicators below, weighted by the highest ranking score and multiplied by 10
Children and older adults (%)	% population aged <10 y or >65 y (2010 US Census Bureau)
Asthma emergency department (visits per 10,000/y)	Spatially modeled, age-adjusted rate of emergency department visits (2007–2009 CEHTP, Office of Statewide Health Planning and Development)
Low birth weight (%)	% births weighing <2500 g (2007–2011 CDPH Vital Statistics)
Educational attainment (%)	% population aged >25 y with < high school education (2007–2011 ACS 5-y estimates, US Census Bureau)
Linguistic isolation (%)	% households in which no one aged ≥ 14 y speaks English "very well" (2007–2011 ACS 5-y estimates, US Census Bureau)
Poverty (%)	% population living below twice the federal poverty level (2007–2011 ACS 5-y estimates, US Census Bureau)
Race/ethnicity (%)	% non-white (2007–2011 ACS 5-y estimates, US Census Bureau)

Note. ACS = American Community Survey; CDPH = California Department of Public Health; CEHTP = California Environmental Health Tracking Program; CARB = California Air Resources Board; CIA = Closed, Illegal, and Abandoned; DTSC = Department of Toxic Substances Control; PM = particulate matter; SWIS = Solid Waste Information System; SWRCB = State Water Resources Control Board.
